# Folic acid tagged nanoceria as a novel therapeutic agent in ovarian cancer

**DOI:** 10.1186/s12885-016-2206-4

**Published:** 2016-03-15

**Authors:** Miriana Hijaz, Soumen Das, Ismail Mert, Ankur Gupta, Zaid Al-Wahab, Calvin Tebbe, Sajad Dar, Jasdeep Chhina, Shailendra Giri, Adnan Munkarah, Sudipta Seal, Ramandeep Rattan

**Affiliations:** Division of Gynecology Oncology, Department of Women’s Health, Henry Ford Hospital, Detroit, MI USA; Advanced Materials Processing and Analysis Center, Nanoscience and Technology Center, Materials Science and Engineering, University of Central Florida, Orlando, FL USA; Wayne State University, Detroit, MI USA; Department of Neurology, Henry Ford Hospital, Detroit, MI USA; Josephine Ford Cancer Institute, Henry Ford Hospital, Detroit, MI USA; College of Medicine, University of Central Florida, Orlando, FL USA

**Keywords:** Folic acid conjugated nanoceria, Ovarian cancer, Nano cerium oxide, A2780, Targeted therapy

## Abstract

**Background:**

Nanomedicine is a very promising field and nanomedical drugs have recently been used as therapeutic agents against cancer. In a previous study, we showed that Nanoceria (NCe), nanoparticles of cerium oxide, significantly inhibited production of reactive oxygen species, cell migration and invasion of ovarian cancer cells in vitro, without affecting cell proliferation and significantly reduced tumor growth in an ovarian cancer xenograft nude model. Increased expression of folate receptor-α, an isoform of membrane-bound folate receptors, has been described in ovarian cancer. To enable NCe to specifically target ovarian cancer cells, we conjugated nanoceria to folic acid (NCe-FA). Our aim was to investigate the pre-clinical efficacy of NCe-FA alone and in combination with Cisplatin.

**Methods:**

Ovarian cancer cell lines were treated with NCe or NCe-FA. Cell viability was assessed by MTT and colony forming units. In vivo studies were carried in A2780 generated mouse xenografts treated with 0.1 mg/Kg NCe, 0.1 mg/Kg; NCe-FA and cisplatinum, 4 mg/Kg by intra-peritoneal injections. Tumor weights and burden scores were determined. Immunohistochemistry and toxicity assays were used to evaluate treatment effects.

**Results:**

We show that folic acid conjugation of NCe increased the cellular NCe internalization and inhibited cell proliferation. Mice treated with NCe-FA had a lower tumor burden compared to NCe, without any vital organ toxicity. Combination of NCe-FA with cisplatinum decreased the tumor burden more significantly. Moreover, NCe-FA was also effective in reducing proliferation and angiogenesis in the xenograft mouse model.

**Conclusion:**

Thus, specific targeting of ovarian cancer cells by NCe-FA holds great potential as an effective therapeutic alone or in combination with standard chemotherapy.

**Electronic supplementary material:**

The online version of this article (doi:10.1186/s12885-016-2206-4) contains supplementary material, which is available to authorized users.

## Background

Epithelial ovarian cancer is the fifth most common cause of cancer-related death in women and the most lethal gynecologic malignancy due to late stage diagnosis and upfront or eventual resistance to chemotherapy. Epithelial ovarian cancer is responsible for approximately 21,290 new cases and 14,180 deaths in 2015 in the United States [1]. Despite new treatment modalities and improvements in diagnostic methods and surgical techniques, only a marginal improvement in survival has been achieved in the last 15 years and most patients will eventually have recurrence and succumb to their disease [2]. Currently, optimal surgical debulking followed by platinum-based chemotherapy is the backbone of the ovarian cancer treatment; however, novel treatment modalities with less toxicity are urgently needed.

Nanotechnology is defined as the science involved in the design, synthesis, characterization, and manufacturing of materials at the nanoscale level [3]. There has been a rapid growth in the nanotechnology field towards the development of nanomedicine products to improve therapeutic strategies against cancer [4]. These nanomedical agents have been shown to improve the pharmacokinetic and pharmacodynamic properties of conventional treatment agents and may enhance the efficacy of existing anticancer compounds with less toxicity [4]. Cerium is a rare earth metal and when combined with oxygen, can adopt a fluorite crystalline lattice structure. Nanoceria (NCe) particles can reversibly bind oxygen and shift oxidation states (Ce^3+^/Ce^4+^) depending on the environment [5]. This interconversion of oxidation states gives rise to a large number of oxygen vacancies on the surface of NCe and is the key property that makes it a potent antioxidant agent. Due to its strong antioxidant properties, we have tested NCe as a therapeutic agent both in vitro and in vivo in ovarian cancer cells and showed that NCe inhibited the reactive oxygen species (ROS), vascular endothelial growth factor induced proliferation and capillary tube formation of endothelial cells. [6]. NCe also attenuated cell migration and invasion without affecting the cell proliferation in ovarian cancer cell lines [6]. Additionally, in vivo treatment of NCe was associated with significant reduction in tumor growth and accompanied by decreased proliferation and attenuation of angiogenesis in a nude mouse model of ovarian cancer. [6]. NCe has also shown anticancer activity in other types of cancers including cutaneous squamous [7], colon [8] and pancreatic cancer [9] models. Recently, it has been demonstrated that NCe in cancer cells associated with acidic conditions prefers to act as a ROS activator, resulting in accumulation of H_2_O_2_ [9]. At acidic pH, NCe becomes an efficient superoxide dismutase mimetic compared to normal pH and converts superoxide radicals to H_2_O_2_. On the other hand, at acidic pH, NCe cannot scavenge H_2_O_2_ and eventually results in accumulation of H_2_O_2_ in cancer cells. This switch from acting as a superoxide dismutase or catalase mimetic to H_2_O_2_ accumulation has been attributed to the acidity dependent surface valence states of NCe (Ce^3+^ or Ce^4+^) [7]. This increased generation of ROS may also be one of the mechanisms of NCe’s antitumor effect.

Folate is a basic component of cell metabolism, DNA synthesis and repair and essential for both normal and tumor cells [10]. Two cellular folate uptake pathways have been identified: transmembrane reduced folate carrier and membrane-found folate receptors (FR) [11]. Interestingly, normal cells utilize reduced folate carrier as a predominant way for folate uptake; however, FR is poorly expressed or absent in normal cells and strongly expressed in cancer cells [11]. FRs capture folate from outside of the cell and the engulfed endosome is transported to the intracellular compartment [10]. Three isoforms of FRs have been identified so far and these receptors have been the focus of cancer therapy. Increased expression of FR-α has been described in various tumor tissues, including ovarian, endometrial and breast cancer [12], and higher FR-α expression was found to be associated with poor chemotherapy response and survival outcome in ovarian cancer [13]. Monoclonal antibodies against FR-α, namely farletuzumab, combined with carboplatinum and taxanes was shown to increase the response rate in platinum-sensitive ovarian cancer patients [14], but in another study in platinum-resistant/refractory patients, combination of farletuzumab with weekly paclitaxel did not reach its endpoint [15]. Not only were monoclonal antibodies against FR- α investigated for treatment, but cellular entry process of folate was also utilized for targeted drug delivery in ovarian cancer by means of linking folate with chemotherapeutic agents. A combined treatment of vintafolide (EC145), a conjugate of folate with desacetylvinblastine monohydrazide, with liposomal doxorubicin was demonstrated to improve progression free survival over standard therapy in a randomized trial of patients with platinum-resistant ovarian cancer [11].

In the current study, we investigated the preclinical efficacy of targeted delivery of NCe by its conjugation to folic acid (NCe-FA) alone and in combination with cisplatinum in inhibiting ovarian cancer growth in vitro and in vivo.

## Methods

### Cell culture

A2780 and C200 were kindly provided by Dr. Thomas Hamilton, Fox Chase Cancer Center, Philadelphia, PA. The cell lines were maintained in Roswell Park Memorial Institute media (HyClone-ThermoScientific, Waltham, MA) supplemented with 10 % fetal bovine serum (BioAbChem) and insulin. Ovarian cancer cell lines, OVCAR3 and SKOV3 were from ATCC (Manassas, VA) and grown according to accompanied instructions.

### Synthesis and characterization of nanoceria-folic acid (NCe-FA)

Cerium oxide nanoparticles were synthesized by using precipitation method. Briefly, Ce(NO)_3_, 6H_2_O (99.999 % pure) were dissolved in deionized water and hydrolyzed using NH_4_OH and pH 9 was maintained [16]. The solution was then stirred for 4 h and the obtained precipitate was then washed with deionized water 3 times. Finally, cerium oxide nanoparticles (NCe) were dried overnight under vacuum at 60 °C. Next, a thin coating of APTMS on the surface of the cerium oxide nanoparticles was then incorporated. This coating provided the amine (−NH_2_) functional group to conjugate folic acid on the surface of the NCe [17]. Folic acid was conjugated to the amine functionalized NCe by using 1-ethyl-3-[3-dimethylaminopropyl] carbodiimide hydrochloride (EDC) and N-hydroxysulfosuccinimide coupling chemistry [17]. Molar ratio of folic acid:NCe-NH_2_: N-hydroxysulfosuccinimide-EDC was 1:1:2.2:11.2. Reaction was carried for 24 h and then washed in both dimethyl sulfoxide and water to remove the EDC, N-hydroxysulfosuccinimide and unbound folic acid. Finally, folic acid conjugated NCe (NCe-FA) were dried under vacuum at 40 °C. NCe-FA was then characterized by using high resolution transmission electron microscopy, X-ray diffraction, X-ray photoelectron spectroscopy and dynamic light scattering. Numbers of the folic acid molecules on top of the 1 nanocrystal were then calculated with help of wet loss from thermogravimetric analysis.

### Nanoparticle uptake studies

NCe, NCe-APTMS and NCe-FA were incubated at different concentrations for 24 h. After incubation cells were washed 3 times using PBS (pH 5) to remove any surface bound nanoparticles. Cells were then collected by scraping and inductively coupled plasma mass spectroscopy was carried out to quantify uptake of NCe as describe in a previous publication [18]. To visualize the uptake of NCe-FA, one million A2780 cells were incubated with NCe-FA, washed and fixed in Trump’s fixative (1 % glutaraldehyde and 4 % formaldehyde in 0.1 M phosphate buffer, pH 7.2) for 24 h. Cell pellet was embedded in Spurr’s resin and thin (90 nm) sections were cut on a Reichert Ultracut E ultramicrotome and placed on 200 mesh copper grids. Pictures were taken using Philips Transmission Electron Microscope (TEM) 208 at the Henry Ford Hospital Electron Microscopy Core.

### ROS estimation

ROS was determined using the membrane-permeable fluorescent dye 6-carboxy2',7'-dicholorohydrofluorescein diacetate (DCFDA) in serum-free medium as described previously [6]. A2780 cells 48 h pretreated with NCe or NCe-FA were treated with 5 μM DCFDA dye and fluorescence was measured at excitation 485 nm and emission 530 nm for various time period from 10 to 60 min using Synergy H1 hybrid reader monochromator system (BioTek, Winooski, VT) [6].

### Proliferation assay

To determine the effect of nanoparticles on proliferation of ovarian cancer cells, 4000 cells per well were plated in 96-well plates in Roswell Park Memorial Institute media containing 10 % (v/v) fetal bovine serum overnight. The next day, the cells were treated with indicated concentrations of NCe or NCe-FA (10–100 μM). Before treatment the NCe and NCe-FA suspension solution was left undisturbed for 30 min of sedimentation, to get rid of the aggregated particles. Solution from the top layer was used for all treatments. Cellular respiration as an indicator of cell number was determined by MTT assay performed at 24, 48 and 72 h post-treatment as described earlier [19]. MTT was purchased from Sigma.

### ApoTox-Glo Triplex assay

To determine the effect of NCe-FA on survival, cytotoxicity and apoptosis of ovarian cancer cells, 4000 cells per well were plated in 96-well plates and treated with indicated concentration of NCe-FA for 48 h. At end of 48 h, ApoTox-Glo Triplex assay kit (Promega; Madison, WI) was used as per the manufacturer’s instructions to assess cell viability and caspase3/7 activity [20]. Briefly, the assay measures all three parameters in the same cells by combining fluorescence based measurement of two differential proteases biomarkers detecting live cells and dead cells. A second reagent containing luminogenic DEVD-peptide substrate for caspase-3/7 and Ultra-Glo™ Recombinant Thermostable Luciferase was added, and the activity measured with a luminometer. BioTek Synergy H1 Hybrid Reader (ThermoScientific) was used to measure the output reads.

### Colony formation assay

A total of 2000 cells were plated in triplicates in 6-well plates and treated with indicated concentrations of NCe or NCe-FA. The cells were allowed to form colonies for up to 2 to 4 weeks. Colonies were stained with MTT and counted as described previously [6].

### Animal studies

#### Ethics statement

All mice were housed and maintained under specific conditions in facilities at Henry Ford Hospital, Detroit, MI. The facilities are approved and inspected by the American Association for Accreditation of Laboratory Animal Care and managed in full accordance with current regulations and standards of the U.S. Department of Agriculture, U.S. Department of Health and Human Services, and the National Institutes of Health. All studies were approved and supervised by the Institutional Animal Care and Use Committee, under the protocol number 1156. Institutional guidelines for the proper and humane use of animals in research were followed. Mice were regularly monitored for any distress once tumor was established and were humanly sacrificed on reaching the end point. Regular health and well-being checks were also performed by Henry Ford Hospital veterinarian staff.

Six-week-old female nude mice were purchased from the National Cancer Institute-Frederick Cancer Research and Development Center (Frederick, MD). A2780 cells at a concentration of 2 × 10^6^/200 μl suspended in PBS were injected into the intraperitoneal cavity (day 0) of 6 to 7-week-old nude mice as described before [21]. Mice were randomized into various groups (*n* = 10). For the first experiment, Group 1 was treated as the control and received PBS intraperitoneally, every third day. Group 2: received NCe treatment at the dose of 0.1 mg/kg body weight, every third day from 3 days postinoculation of tumor cells till the end of the study. Group 3 received NCe-FA treatment at the dose of 0.1 mg/kg body weight, every third day from 3 days postinoculation of tumor cells till the end of the study. For the second experiment (*n* = 8 per group), Group 1 acted as the control and PBS was injected intraperitoneally, every third day. Group 2 received NCe-FA treatment at the dose of 0.1 mg/kg body weight, every third day from 3 days postinoculation of tumor cells till the end of the study. Group 3 received NCe-FA treatment along with cisplatinum treatment at 4 mg/kg of body weight once a week intraperitoneally. Group 4 received 3 cisplatinum treatments alone at the dose of 4 mg/kg of body weight once a week. After tumor induction, mice were monitored daily for signs of any distress. Regular health checks are also performed by the veterinarian staff. Mice were humanly sacrificed when the tumor burden reached the mandate weight in untreated mice at 4 weeks. Tumors were fixed in formalin for sectioning. Blood was collected in heparin-coated tubes to obtain plasma. Liver, kidney, heart and spleen from all animals were formalin fixed and processed. One tumor and organ slide from each mouse was stained with hematoxylin and eosin.

### Toxicity assays

Blood was collected in heparin-coated tubes immediately after mouse euthanasia. Plasma was isolated from the blood and subjected to analysis of a panel of liver function tests (aspartate aminotransferase, alanine aminotransferase and albumin) and kidney function tests (creatinine, urea and albumin) as described before [22]. All assays were performed using kits from Bioassay Systems following the manufacturer’s instruction (Hayward, CA).

### Tumor score

Tumor nodules morphology and count were identified grossly in various organs. These included the liver, kidneys, bowel and peritoneum and described before [21]. A score of 0 was given for no nodule in the organ; 1 for 1 nodule; 2 for 2 to 5 nodules and score 3 for more than 5 nodules observed per organ. Two individuals performed the scoring in a blinded manner and included a gynecology oncology fellow (ZW).

### Immunohistochemistry

The tumors excised from mice were fixed in 10 % paraformaldehyde for 48 h and paraffin-embedded. Consecutive sections of 4 microns thick were cut and processed for hematoxylin and eosin staining and immunohistochemistry for Ki-67 (used at 1:100; catalog No: M7240, Dako) and cleaved caspase-3 (used at 1:200, catalog No: 9661, Cell Signaling Technology). Anti-4 Hydroxynonenal (4-HNE) antibody (catalog No: ab46545, used at 1:100) and CD31 antibodies were from Abcam (used at 1: 200, catalog No. 28364). Vimentin (used at 1:100, catalog No: M0725) antibody was obtained from Dako and staining was performed according to the manufacturer’s instruction. The positive cells stained brown. The slides were examined under a light microscope, and representative pictures were taken from a minimum of 4–5 slides from each group [23]. The quantification of the stain intensity was performed by assigning a score of 0–1 for no or weak stain; 2 for moderate stain and 3 for strong stain. All slides were examined in a blinded manner.

### Statistical analysis

Data were statistically analyzed using the Graph Pad Prism software (GraphPad Software Inc, La Jolla, CA) using a combination of t-test and analysis of variance methods.

## Results

### Synthesis and characterization of Cerium - Folic acid (NCe-FA)

**Fig. 1:**
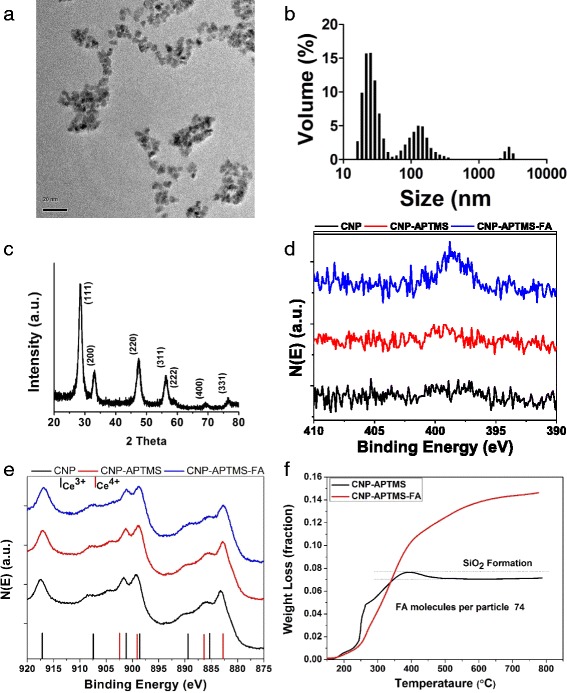
Detailed characterization of synthesized NCe-FA. **a** High resolution transmission electron microscopy image of the NCe-FA showed particles sized 8–10 nm. **b** The hydrodynamic size of the nanoparticles showed slight agglomeration of the particles in cell culture medium, 76 % of particles in the range between 20 and 70 nm. **c** X-Ray T spectrum shows the crystalline property of the nanoparticles. **d** X-ray photoelectron spectroscopy spectra of nitrogen 1S of NCe, NCe-EPH and NCe-EPH-FA confirms the presence of folic acid on the surface of the nanoparticles. **e** X-ray photoelectron spectroscopy spectra of Ce3d of NCe, NCe-APTMS and NCe-APTMS-FA showed no significant change in surface chemistry (Ce^3+^/Ce^4+^) due to modification of the surface. **f** Thermogravimetric analysis weight loss spectra for NCe-APTMS and NCe-APTMS-FA showed presence of folic acid on the surface of the NCe nanoparticle

Folic acid-coated nanoparticles were thoroughly characterized. High-resolution transmission electron microscopy images show the size of the NCe-FA is approximately 10 nm (Fig. 1a). NCe-FA suspension was also analyzed using dynamic light scattering to identify the nanoparticles size or extent of agglomeration in complete cell culture medium (Eagle’s minimum essential medium with 10 % fetal bovine serum). Figure 1b shows the histogram for the NCe-FA size distribution. Cell culture medium was used for dynamic light scattering measurement as ion and protein rich medium is known to influence nanoparticles aggregation and all cell culture experiments were carried out in a similar environment [24]. The hydrodynamic size of NCe-FA nanoparticles was found as approximately 30 nm with a small percentage (26 %) having agglomerated particles of size 150 nm. A slight increase in size (~30 nm) may indicate formation of loose agglomeration and absorption of protein/ions increase the hydrodynamic size. The surface charge of the NCe-FA was also analyzed and found to be 25 mV. X-ray diffraction data confirmed the microcrystalline nature of the particles (Fig. 1c). X-ray photoelectron spectroscopy spectra of nitrogen 1S revealed folic acid presence on the surface of the nanoparticles (Fig. 1d). Characteristic peaks of NCe-FA N1s spectra was deconvoluted and following peaks were assigned 397.25 eV for C-N = C, 398.6 eV for N-H and 400.8 eV for N-C = O, which confirmed the presence of folic acid on the surface of the nanoparticles (Additional file 1: Fig. S1A). Absence of nitrogen signal for APTMS-NCe might be due to the presence of a very thin coating and high signal versus noise ration of the instrument. X-ray photoelectron spectroscopy spectra of Ce 3d of NCe, 3-aminopropyl-trimethoxysilane (APTMS)-NCe and NCe-FA revealed no significant changes in the surface oxidation state during the reaction/surface modification of ceria with folic acid (Fig. 1e). X-ray photoelectron spectroscopy spectrum was further deconvoluted to determine the surface oxidation state on the surface of NCe-FA, as discussed elsewhere (Additional file 1: Fig. S1B) [22]. Surface oxidation states of NCe-FA play an important role in determining NCe’s redox activity. As mentioned earlier, redox activity of NCe is due to the presence of Ce^3+^ and Ce^4+^ on the surface of the nanoparticles [25]. The Ce^3+^/Ce^4+^ oxidation ratio for NCe-FA was 47.4 %. Weight loss results from thermogravimatric analysis (Fig. 1f) were used to calculate approximate numbers of folic acid ligands on the surface of each nanoparticles (assuming NCe diameter is 10 nm according to the transmission electron microscopy observation) where NCe-APTMS weight loss data were used as a baseline (detailed calculations are shown in the Additional file 2). Weight loss data for bare NCe, APTMS-NCe and NCe-FA are shown in Additional file 1: Fig. S1C. Approximate 74 folic acid molecules were conjugated on the surface of each NCe-FA particle.

### NCe-FA showed a higher internalization rate compared to NCe

**Fig. 2:**
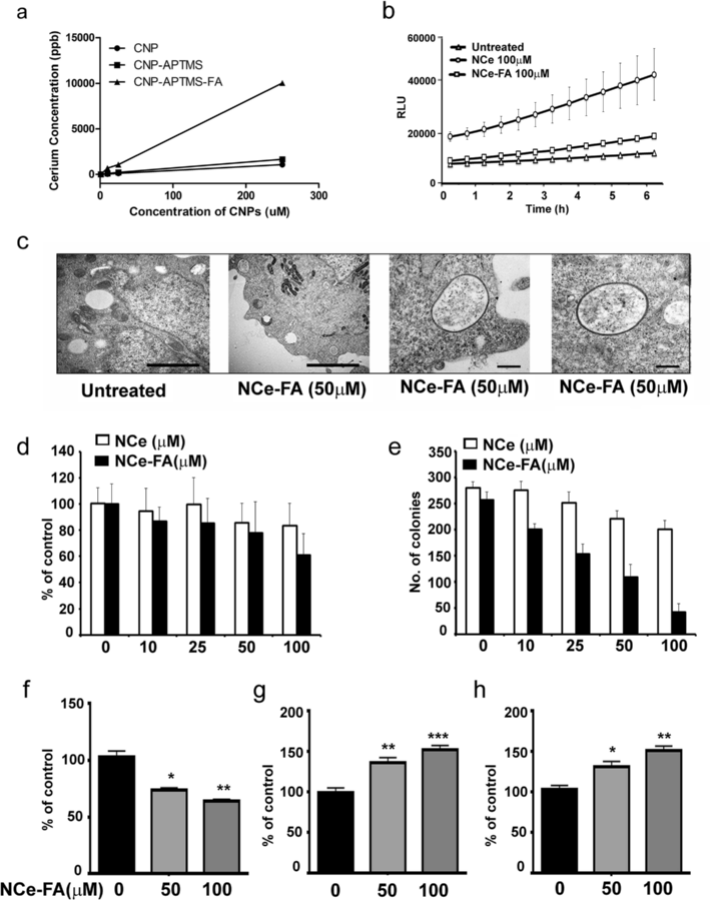
NCe-FA internalizes and inhibits ovarian cancer cell proliferation. **a** Increasing concentrations of the various nanoparticles measured inside the cancer in a dose-dependent manner. **b** ROS generated by NCe and NCe-FA over a period of time. **c** Representative TEM pictures of cells with vacuoles containing NCe-FA particle. Panels 1 and 2 show images from control and NCe-FA treated cells at a magnification of 18000. Panels 3 and 4 show magnified images of the vacuoles containing NCe-FA particles (magnification 28000). **d** Decreasing number of cells under NCe-FA treatment compared to NCe alone at 48 h as seen by MTT assay. **e** Decreasing number of colonies formed under NCe-FA treatment compared to NCe alone as observed by clonogenic survival assay. **f** Decreased protease-based cell viability under NCe-FA treatments at 48 h. Values are represented as percent of control, with control taken as 100 % viable cells. **g** Increased protease-based cell cytotoxicity under NCe-FA treatments at 48 h. Values are represented as percent of control. **h** Caspase 7/3 activity under NCe-FA treatments at 48 h, with values represented as percent of control. *p ≤ 0.05, **p ≤ 0.01, ***p ≤ 0.001 compared to control cells

Uptake of NCe-FA by the ovarian cancer cells was assessed by measuring the amount of NCe-FA inside the cells by mass spectroscopy. Cells were treated with NCe alone, functionalized NCe with amine (CO2-NH_2_) by using APTMS (referred to as NCe) [26] and functionalized NCe-FA at a dose range of 0–300 μM. After 24 h, cells were extensively washed to remove excess or any surface-attached nanoparticles. Cell pellet was subjected to mass spectroscopy to determine the amount of nanoparticles inside the cells in terms of parts per billion. A dose-dependent intake inside the cells was observed with the conjugation of folic acid with the NCe particles (Fig. 2a). Further we used TEM to visualize the internalized NCe-FA. In treated A2780 cells NCe-FA particle agglomerates could be observed in distinct vacuoles (2c, second panel), while no such vacuoles could be detected in untreated cells (2c, second first panel). Magnified images of vacuoles are shown in panels three and four (Fig. 2c). Thus, folic acid conjugation significantly increases the uptake of the cerium nanoparticle by the cancer cell.

### NCe-FA inhibits ROS in A2780 cell line

Recent data point toward an important role for oxidative stress in the etiology of ovarian cancer as ROS accumulation plays a significant role in the initiation and progression of ovarian cancer [27, 28]. NCe has been shown to act as a free radical scavenger by inhibiting ROS in ovarian cancer by us and others [29–31]. Acidic extracellular pH is a major feature of malignant tissue, which might be secondary to lactate secretion from anaerobic glycolysis [32, 33]. It was demonstrated that the NCe nanoparticle aids in generation of ROS in cancer cells [9]. We observed that an acidic NCe significantly increased ROS with time (Fig. 2b) in A2780 measured by using DCFDA dye followed by fluorescence reading kinetics up to 6 h. To investigate if tagging of folic acid had any effect on its ROS generating property, we examined the effect of NCe-FA on ROS generation. Interestingly, NCe-FA treatment also increased ROS generation compared to control cells, but at significantly lower levels, compared to NCe particles (Fig. 2b). This indicates that conjugation of folic acid may be compromising the redox modulating capabilities of NCe.

### NCe-FA inhibits ovarian cancer growth in vitro

Our previous data with NCe had shown no effect on proliferation of ovarian cancer cells [6]. Therefore, we aimed to determine if NCe-FA conjugation has an effect on the growth of ovarian cancer cells in vitro due to its increased cellular uptake. To investigate this, A2780 were treated with various concentrations of NCe-FA (10–100 μM) and cellular respiration as an indicator of viable cell number was determined by 3-(4,5-dimethylthiazol-2-yl)-2,5-diphenyltetrazolium bromide (MTT) assay [19]. NCe treatment had no significant effect on the proliferation of A2780, consistent with our previous results (Fig. 2d). However, NCe-FA showed significant inhibition in viable cells in A2780 cells (Fig. 2d, black bars). More effective inhibition was observed in the colony formation assay, where NCe-FA significantly inhibited the colony forming units in A2780 (Fig. 2e, black bars). Similar inhibition of viability and colonogenic survival by NCe-FA in other ovarian cancer lines: SKOV3, OVCAR 3 and C200 was also seen (Additional file 3: Fig. S2). To further validate the reduction in cell number by NCe-FA, we assessed the cell viability, cytotoxicity and caspase7/3 activity simultaneously [20], in A2780 after 48 h of NCe-FA treatment. NCe-FA treated cells showed significant decreased cell viability, increased cell death and caspase 7/3 activation (Fig. 1f-h). These data suggest that by tagging folic acid we enabled NCe to decrease cell variability, possibly due to its increased internalization resulting in apoptosis.

### NCe-FA inhibits ovarian cancer in vivo

**Fig. 3:**
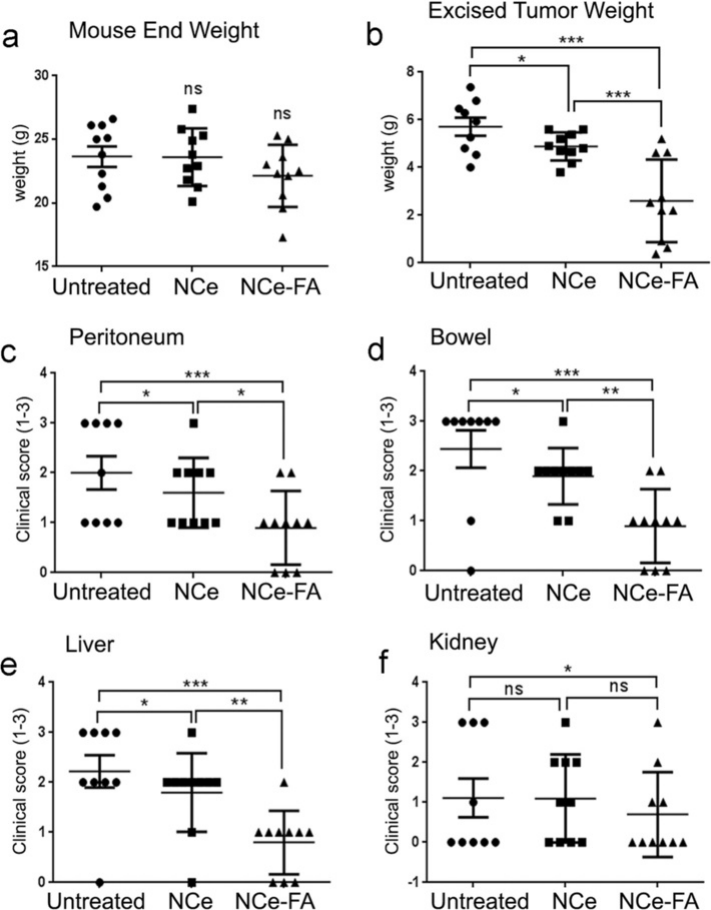
NCe-FA inhibits A2780 ovarian tumor growth in vivo. **a** Each mouse weight at the time of sacrifice at 4 weeks post-tumor inoculation. **b** Weight of the tumor mass excised from each mouse of the respective group. Clinical score enumerated as the number of tumor nodules observed by naked eye at each organ site of the **c** peritoneum, **d** bowel, **e** liver and **f** kidney. A score of 0 was given for no nodules in the organ; 1 for 1 nodule; 2 for 2 to 5 nodules and score 3 for more than 5 nodules observed per organ

Preclinical evaluation of NCe-FA treatments was done in A2780 xenografts bearing nude mice that were treated with either vehicle PBS or NCe (0.1 mg/kg body weight) or NCe-FA (0.1 mg/kg body weight). Mice treated with NCe-FA reflected slower tumor growth as seen by lower mice weights compared to NCe alone and vehicle treated mice (Fig. 3a). Measurement of excised tumor weight showed that NCe-FA was more effective in inhibiting tumor growth (Fig. 3b), indicating that conjugation of folic acid potentiated the efficacy of NCe alone.

The injected cancer cells also homed to the ovary and formed ovary associated tumors, along with spread to the other organs. Ovary associated tumors were excised individually, examined and measured. The pictograph in Additional file 4: Fig. S3 shows that NCe-FA associated ovary tumors were less in size. Also, involvement of only 1 ovary was associated more with NCe-FA treated mice, with some mice showing no ovary involvement at all. This indicated that NCe-FA is effective in reducing the ovary-associated tumor spread.

### NCe-FA treated mice had lower tumor burden compared to NCe and vehicle groups

Tumor burden at vital organ sites was enumerated by assigning a score of 0 if no tumor nodules were observed; 1 for 1 nodule; 2 for 2 to 5 nodules and a score of 3 for more than 5 nodules [21]. All organs examined (peritoneum, bowel, liver and kidney) from the NCe-FA treated group showed a significantly lower score compared to vehicle and NCe treated mice (Fig. 3c–f). The greatest difference was observed in the kidney and liver scores (Fig. 3e, f), indicating that NCe-FA has the potential to contain metastatic spread of ovarian cancer, which could be in part due to restriction of tumor size.

### NCe-FA treated mice did not show any signs of vital organ toxicity

We determined the cellular toxicity of NCe-FA on liver, heart, spleen, kidney and lungs. Formalin fixed organs from untreated and NCe-FA treated mice at the end of the study were stained for hematoxylin and eosin. Examination of the sections revealed that the morphology of all organs from treated and untreated mice appeared to be normal and there were no findings suggestive of tissue necrosis (data not shown). No significant difference in the plasma levels of liver function (aspartate aminotransferase, alanine aminotransferase, and albumin) and kidney function tests (creatinine, urea, albumin and uric acid) collected from untreated and treated mice was observed, and they were all within normal limits in both groups (Additional file 5: Fig. S4). Taken together, these data showed that NCe-FA treatment twice a week for 4 weeks at the dose of 0.1 mg/kg is safe and not associated with any abnormal physiological abnormalities or cytotoxicity.

### NCe-FA enhances cisplatinum's tumoricidal effect

**Fig. 4:**
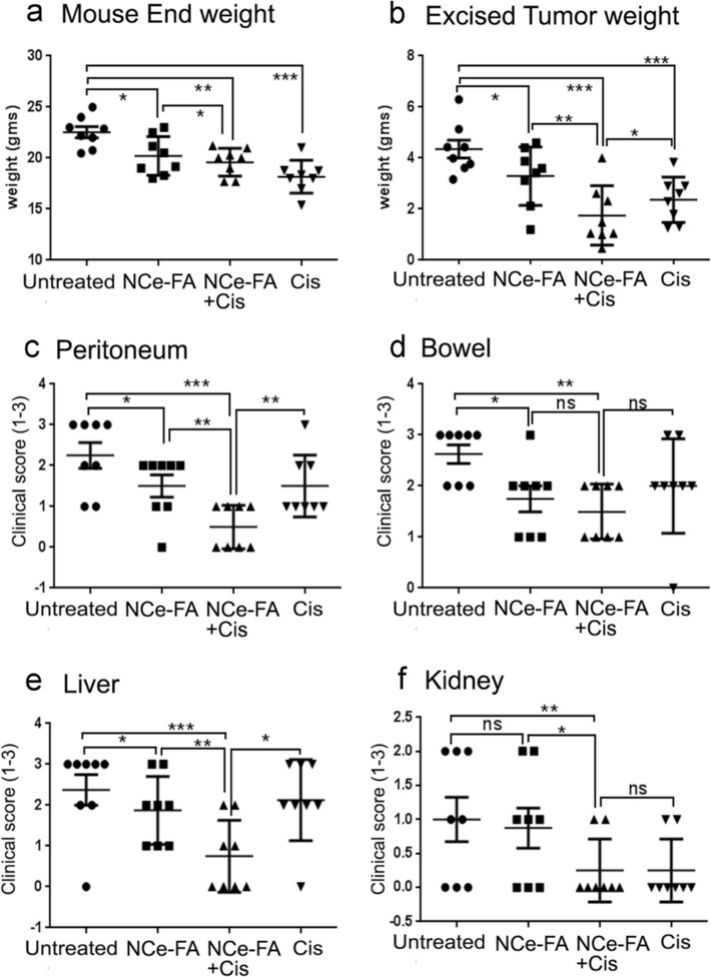
NCe-FA enhances cisplatin toxicity in A2780 ovarian tumors in vivo. **a** Each mouse weight at the time of sacrifice at 4 weeks post-tumor inoculation. **b** Weight of the tumor mass excised from each mouse of the respective group. Clinical score enumerated as the number of tumor nodules observed by naked eye at each organ site of the **c** peritoneum, **d** bowel, **e** liver and **f** kidney. A score of 0 was given for no nodules in the organ; 1 for 1 nodule; 2 for 2 to 5 nodules and score 3 for more than 5 nodules observed per organ

We investigated if combination of NCe-FA with cisplatinum will be more effective in inhibiting ovarian cancer growth. A2780 xenografts were treated with NCe-FA (0.1 mg/kg body weight) as before, with or without cisplatinum (4 mg/kg body weight). Vehicle and cisplatinum alone treatments served as comparative controls. While NCe-FA treated mice had lower weights at the end of 4 weeks, cisplatinum treatments caused a significant decrease in tumor weight, as expected (Fig. 4a). Excised tumor mass was the lowest in the combination group (Fig. 4b), even though the cisplatinum alone group had lower body weights. Enumeration of tumor burden and spread by clinical score in all vital organs [21], showed NCe-FA/cisplatinum combination to have reduced tumor burden at peritoneum and liver (Fig. 4c, e). Tumor scores at bowel and kidney were significantly less in the combination treatment group compared to the NCe-FA group alone, but they did not achieve significance compared to the cisplatinum group alone (Fig. 4d, f). This demonstrates that the decreased tumor score observed in the NCe-FA treatment group was further lowered by cisplatinum combination (Fig. 4c–f). Thus, combination of NCe-FA with cisplatinum is an effective strategy to inhibit ovarian tumor growth in vivo.

### NCe-FA and combination with cisplatinum reduced the proliferation and angiogenesis

**Fig. 5:**
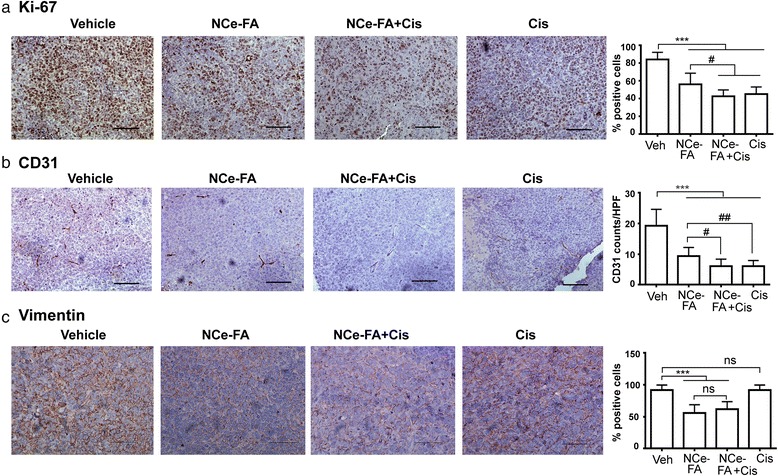
NCe-FA inhibits proliferation and angiogenesis of ovarian tumors in vivo. **a** Representative Ki-67 staining of the tumor tissue, indicating proliferating cells, from various treatment groups (200×). The counts of positive Ki-67 cells from 5 high powered fields (×400) in 3 different xenografts from each group is presented as a bar graph. ***p < 0.001, #*p* < 0.05. **b** Representative CD31 staining of the tumor tissue representing vessel formation from the various treatment groups (200×). Number of positive staining vessels were counted per field from 5 high powered fields (×400) in 3 different xenografts from each group and is presented as a bar graph. **c** Representative vimentin staining of the tumor tissue from the various treatment groups (400×). Cells with positive membrane stain were counted per field from 5 high powered fields (×400). ***p < 0.001; ns, non-significant

In order to ascertain the proliferation of cancer cells, immunohistochemical analysis of Ki67 was performed. Enumeration of Ki-67 positive cells counted over 5 high power fields of 5 sections from each group showed significantly less Ki-67 positive cells in NCe-FA treated xenografts compared to the untreated group (expressed in percentages; Fig. 5a), indicating that less number of cells were proliferating under NCe-FA treatment. The proliferation was further decreased when NCe-FA was combined with cisplatinum. Based on these results, we suggest that NCe-FA has the ability to restrict ovarian tumor growth in vivo due to decreased proliferation of ovarian cancer cells. Since NCe was shown previously by us to inhibit angiogenesis by targeting endothelial specifically, we also performed CD31 staining to analyze angiogenesis in the conjugated groups. CD31 staining of NCe-FA and NCe-FA and cisplatinum treated A2780 tumors demonstrated lower numbers of CD31 positive microvessels (Fig. 5b) compared to the untreated group. However, there was no difference between the NCe-FA and cisplatinum only groups. Since we have previously observed NCe to inhibit migration and invasion of ovarian cancer cells, we stained for markers of epithelial-mesenchymal transition (EMT) to observe if NCe-FA has any effect on the EMT process in vivo. Vimentin, a marker for mesenchymal phenotype of the cells and one of the most common markers of EMT [34], was expressed highly on the membrane of the untreated tumors. NCe-FA treated tumors showed significantly reduced number of positively stained cells and the intensity of the stain alone and in combination with cisplatin (Fig. 5c). Cisplatin by itself did not affect vimentin expression. We also stained for epithelial-Cadherin, but could not detect any expression in the treated or untreated tumor section (data not shown). These findings suggest that NCe-FA/cisplatinum is effective in reducing angiogenesis and EMT markers in A2780 ovarian cancer xenograft model.

### Combination of cisplatinum with NCe-FA increased cisplatinum's apoptotic properties

**Fig. 6:**
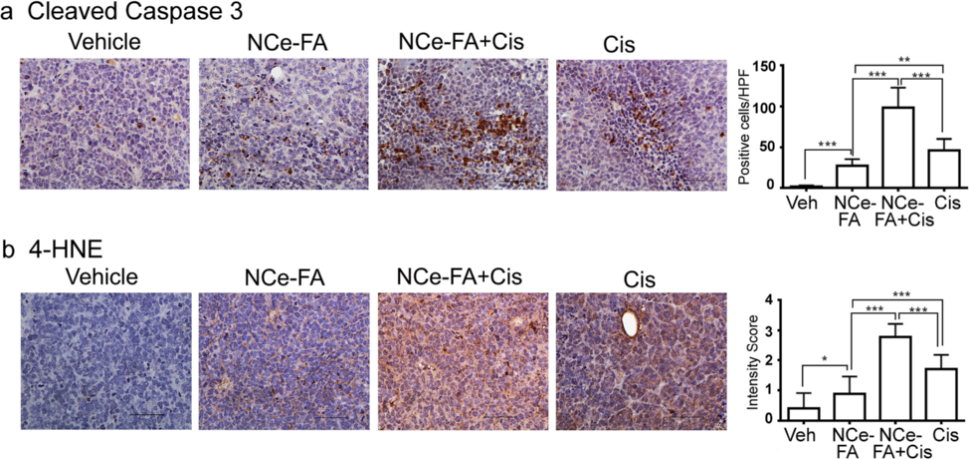
NCe-FA enhances apoptosis and ROS in combination with cisplatinum. **a** Representative caspase-3 staining of the tumor tissue, indicating cells undergoing apoptosis and **b** 4 hydroxynonenal, indicating presence of oxidative stress, from various treatment groups (200×). Expression was visualized as a positive brown stain. Each stained picture is representative of at least 5 different fields examined from 3 individual slides stained per group. Quantification of the stain was performed on a scale of 0–3; 0–1 for no or weak stain; 2 for moderate stain and 3 for strong stain. Average score is represented as a respective bar graph. *p < 0.001, *p < 0.05

Cisplatinum is a chemotherapy agent that forms a platinum complex, and once it enters the cell, binds to DNA, which causes DNA crosslinks as well as oxidative stress and causes cells to undergo apoptosis [35, 36]. Cleaved caspase-3 is an apoptotic marker and plays a key role in apoptotic cell death [37]. The combination of NCe-FA and cisplatinum had the highest caspase-3 among all of the groups (Fig. 6a). Cisplatinum alone showed higher activation of caspase-3 compared to NCe-FA alone. Thus, combination of NCe-FA and cisplatinum significantly increased apoptosis in ovarian cancer cells.

### Modulation of oxidation status in vivo

Since NCe particles function through the modulation of their oxidation states, we also observed ROS production in vitro by NCe-FA (Fig. 2b). Oxidation status in the tumor tissue was estimated by 4-HNE immunostaining, a commonly used marker for lipid peroxidation and oxidative stress [38]. NCe-FA treated tumors stained positive for oxidative stress compared to the control group (Fig. 6b). Cisplatinum treated tumors showed higher expression of 4-HNE compared to the control, in agreement with the ROS producing reports of cisplatinum [39–41]. Tumors treated with the combination of cisplatinum and NCe-FA exhibited the highest degree of oxidative stress (Fig. 6b). This suggests that NCe-FA exhibits oxidant property in vivo, probably due to the different tumor environment present, and could be one of the mechanisms behind its elevated antitumor activity in combination with cisplatinum.

## Discussion

In recent years, there has been a tremendous amount of data accumulation and understanding of tumor biology that could cause a paradigm shift in the treatment of cancer. Conventional chemotherapy still constitutes one of the main strategies for cancer treatment; however, it lacks the specificity against cancer cells and is associated with significant toxicity. Nanotechnology has recently been added to the treatment armamentarium with the aim to expand the horizons of cancer treatment. Nanoparticles have been shown to have a potential for early detection of cancer [42], effective drug delivery that preferentially accumulates the drugs in tumor cells [43] and potentiate the dose deposit selectively in irradiated tissues [44]. In the current study, we showed that cerium nanoparticles conjugated with folic acid had a better cellular uptake as folic acid increased the concentration of NCe in ovarian cancer cells compared to NCe alone, resulting in better tumor inhibition and further enhancement of the tumoricidal effect of cisplatinum.

Cancer cells are highly dependent on folate for replication, and they even develop their own receptors that are not overexpressed on noncancerous cells, which makes it an excellent avenue for targeted anticancer treatment [45, 46]. We previously showed that NCe has significant anticancer effect on ovarian cancer cell lines secondary to its antioxidant and antiangiogenic properties [6]. We exploited this differential expression of folic acid receptors on cancer and somatic cells and demonstrated that conjugation of NCe with folic acid potentiated the NCe's therapeutic effect on ovarian cancer. One of the mechanisms for the higher efficacy might be higher intracellular accumulation of folic acid tagged particles. NCe-FA had significantly increased uptake by the cells, indicating that the conjugation with folic acid indeed enabled NCe to specifically target the cancer cells.

Tumor angiogenesis is essential for delivering oxygen and other nutrients to the growing tumor and is characterized by either excess production of pro-angiogenic signals or lack of angiogenesis inhibitors [47–49]. Recently, inhibition of tumor angiogenesis has become a clinical anticancer strategy and only a few new drugs have been approved by the U.S. Food and Drug Administration for ovarian cancer since 2006, including an anti-angiogenic agent, bevacizumab [50]. In our previous study, NCe was found to have significant antiangiogenic properties that is independent of VEGF production in vitro and in vivo*.* We speculated that this might be secondary to inhibition of new vessel formation by targeting endothelial cells. In the current study, we examined the microvessel density in each group and found a significant reduction in formation of new vessels in NCe-FA and NCe-FA/cisplatinum treated groups compared to the untreated group as evident from decreased expression of CD31, a marker for endothelial cells. However, the NCe-FA and cisplatinum combination and cisplatinum alone groups showed similar CD31 density despite the fact that a lower tumor burden score was achieved with NCe-FA and cisplatinum treatment. Although the vessel density was similar, there might be a possibility that the integrity of the vessels may be more compromised in the combination group, resulting in nonfunctional vasculature, hence more reduced tumor growth. Additionally, NCe-FA also reduced the expression of the EMT marker, vimentin [34], indicating its ability to maybe limit ovarian tumor metastasis. This supports our observation of reduced tumor nodules on the abdominal organs in the treated groups.

NCe particles have a dual effect on ROS generation that is mainly dependent on the pH of the environment [7]. In the previous study, we showed that NCe (with higher Ce^3+^; ~62 %) has significant antioxidant properties and decreased the ROS generation in A2780 cell lines in vitro. However, in the current study, a different formulation of NCe (with lower Ce^3+^ [~24 %] as compared to the study published earlier) and NCe-FA conjugation increased the ROS. This might be secondary to the pH of the cancer cells. Wason et al. suggested that NCe acts primarily as a producer of H_2_O_2_ in (acidic) a cancer environment and a scavenger of H_2_O_2_ in (neutral) normal tissues [9]. In particular, NCe converts superoxide radicals to H2O2; however, in an acidic condition pH suppress NCe catalase mimetic activity, which causes accumulation of H_2_O_2_ within the cells. In the presence of Fe ions, H_2_O_2_ can go through Fenton mediated reaction and might generate reactive radicals. Chemotherapy agents, such as anthracyclines and platinum complexes, also generate ROS and cause chemotherapy associated oxidative stress [51]. This eventually decreases cell proliferation [51]. The combination of NCe-FA and cisplatinum showed the strongest positive expression of 4 hydroxynonenal (oxidative stress marker) as they both increased the ROS production and eventually caused oxidative stress. These data are in accordance to the previous publication where NCe was shown to induce ROS generation in squamous carcinoma cells [7] and pancreatic cancer cells [9].

## Conclusion

In conclusion, conjugation of nanoparticle cerium oxide with folic acid has significant in vivo and in vitro antitumor activity in ovarian cancer and combination treatment with cisplatinum has enhanced this activity. In the future, using novel technologies including nanomedicine particles and modifying them in a more tumor targeted fashion given with or without conventional chemotherapy would provide better clinical outcomes with less toxicity and offer a superior treatment modality and hope to our patients with ovarian cancer.

## Additional files

Additional file 1: XPS spectra of the nanaoparticles. (TIF 10 MB) Additional file 2: Calculations to determine the wight of nanoparticles. (DOCX 13 kb) Additional file 3: NCe-FA inhibits growth of various ovarian cancer cell lines. (TIF 9 MB) Additional file 4: NCe-FA inhibits growth of ovary associated tumors in vivo. (TIF 12 MB) Additional file 5: NCe-FA treatment did not result in any toxocity. (TIF 8 MB)

## Abbreviations

DCFDA: 6-carboxy 2′, 7′-dichlorodihydrofluorescein diacetate
EDC: 1-ethyl-3-[3-dimethylaminopropyl] carbodiimide hydrochloride
FR: folate receptors
MTT: 3-(4,5-dimethylthiazol-2-yl)-2,5-diphenyltetrazolium bromide
NCe: nanoceria
NCe-FA: folic acid conjugated with nanoceria
PBS: phosphate-buffered saline
ROS: reactive oxygen species
