# Supplementary figures and images for: Folic acid tagged nanoceria as a novel therapeutic agent in ovarian cancer
Source: BMC Cancer. 2016 Mar 15;16:220. doi: 10.1186/s12885-016-2206-4 (PMC4791781; doi:10.1186/s12885-016-2206-4)

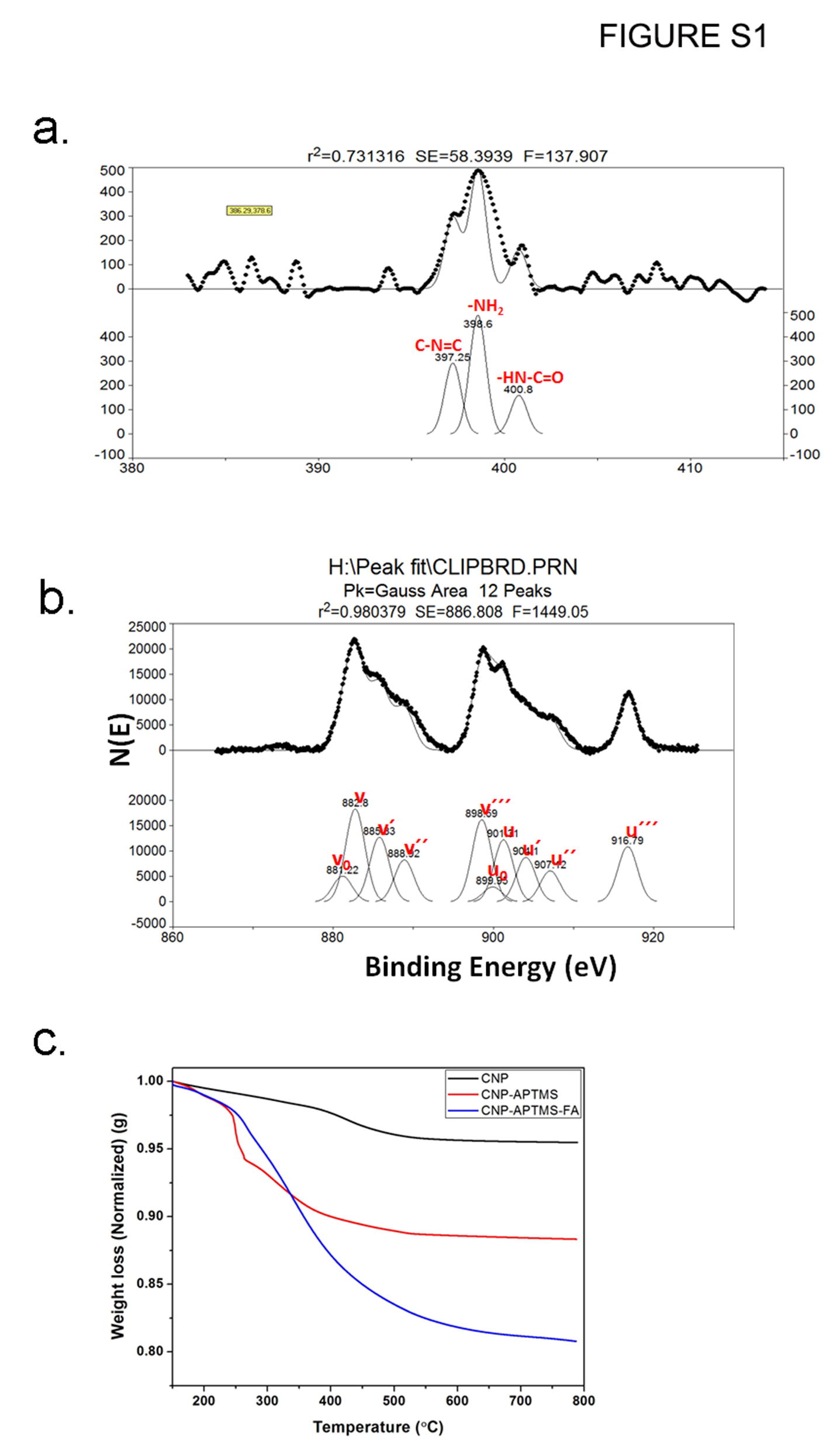

Supplement: Additional file 1: — XPS spectra of the nanaoparticles. (TIF 10 MB) [file 12885_2016_2206_MOESM1_ESM.tif]

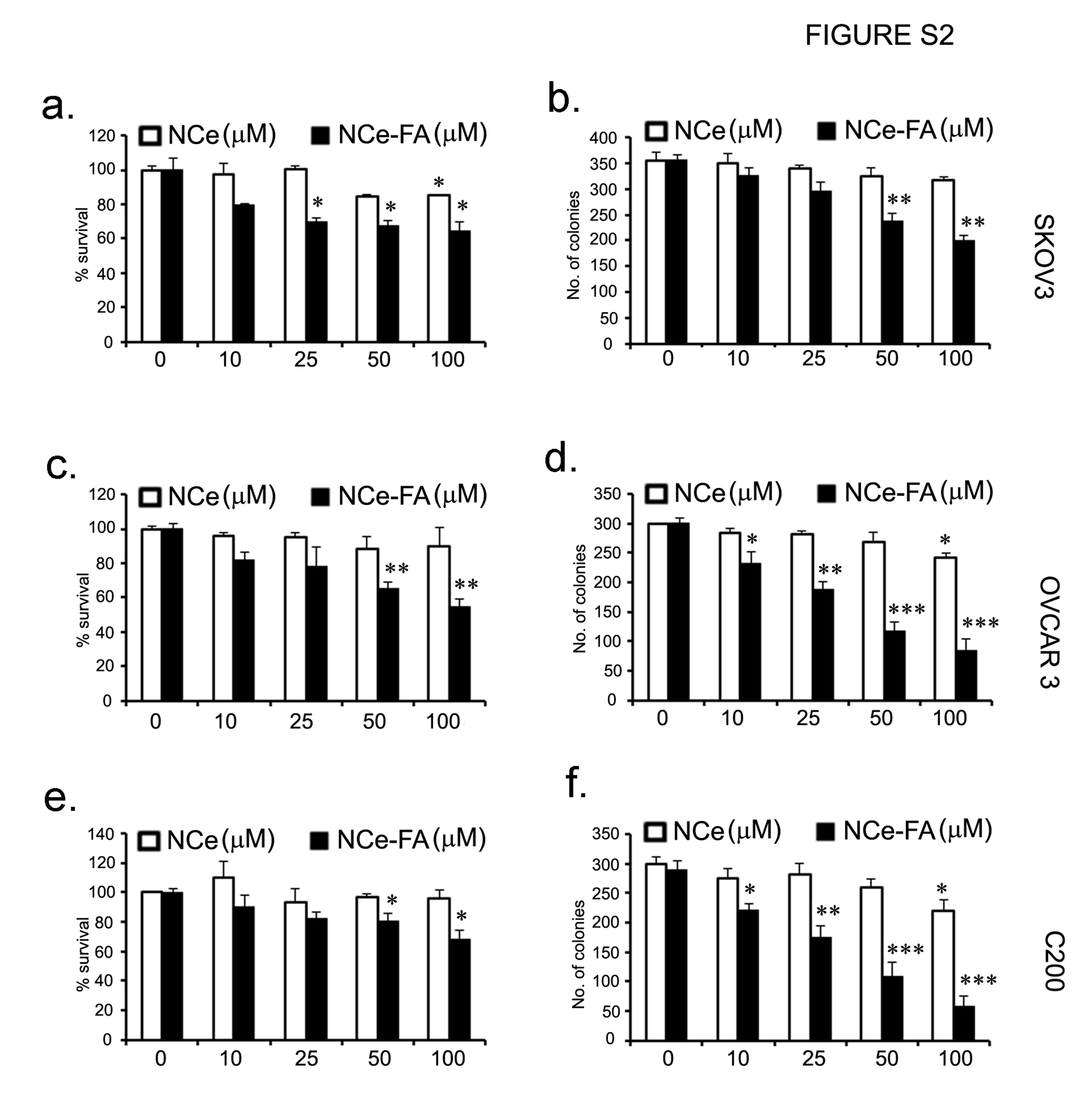

Supplement: Additional file 3: — NCe-FA inhibits growth of various ovarian cancer cell lines. (TIF 9 MB) [file 12885_2016_2206_MOESM3_ESM.tif]

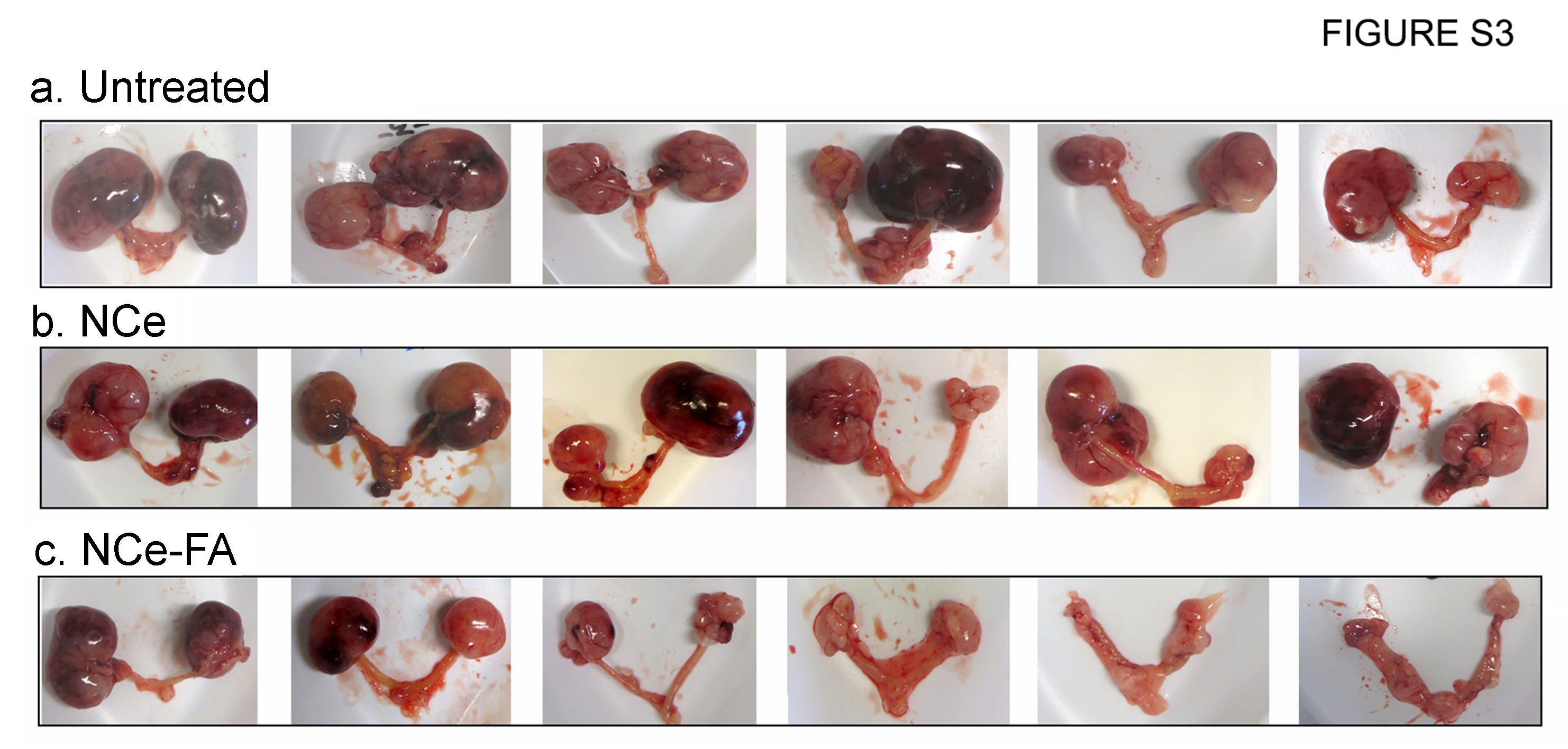

Supplement: Additional file 4: — NCe-FA inhibits growth of ovary associated tumors in vivo. (TIF 12 MB) [file 12885_2016_2206_MOESM4_ESM.tif]

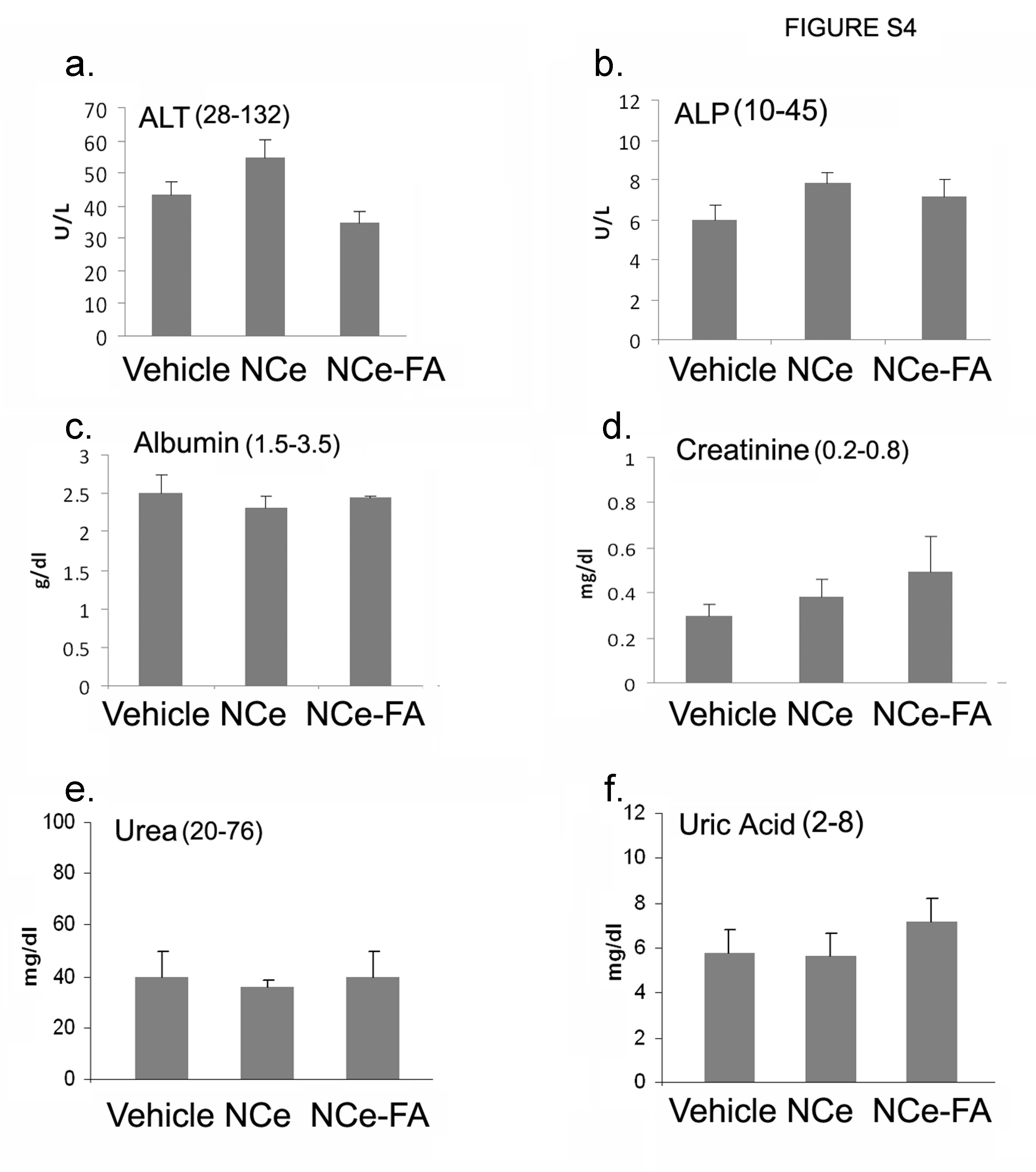

Supplement: Additional file 5: — NCe-FA treatment did not result in any toxocity. (TIF 8 MB) [file 12885_2016_2206_MOESM5_ESM.tif]
