# Supplementary material for: Folic acid tagged nanoceria as a novel therapeutic agent in ovarian cancer
Source: BMC Cancer. 2016 Mar 15;16:220. doi: 10.1186/s12885-016-2206-4 (PMC4791781; doi:10.1186/s12885-016-2206-4)
Supplement: Additional file 2: — Calculations to determine the wight of nanoparticles. (DOCX 13 kb) [file 12885_2016_2206_MOESM2_ESM.docx]

**Supplemental information**

Numbers of particles of a given weight of the nanoparticles were calculated using the following equations.

**NCe particle calculation**

Weight loss = initial weight - final weight

Weight loss percentage = (weight loss/initial weight)*100

Density of NCe = 7.13 gm/cm^3^

Total volume of NCe = final weight/density

Volume of NCe core = $\frac{4}{3}\pi{R_{1}}^{3}$ (assuming CNP particle size of 10 nm according to transmission electron microscopy micrograph)

No. CNP particles = total volume of CNP/volume of CNP core

**Number of molecules on APTMS**

Weight loss = initial weight - final weight

Weight loss percentage_total_ = (weight loss/initial weight)*100

Weight loss percentage_APTMS_ = (weight loss percentage)_total_ – (weight loss percentage)_CNP_

Weight of APTMS molecules = (weight loss percentage_APTMS_ x initial weight)/100

Total APTMS molecules in sample = (weight of APTMS molecules/(molecular weight of molecule + 32))*N_A_

APTMS has one Si atom per molecule

No. molecules per particles = (total APTMS molecules in sample)/(No. CNP particles)

**Number of molecule on folic acid**

Weight loss = initial weight - final weight

Weight loss percentage_total_ = (weight loss/initial weight)*100

Weight loss percentage_FA+SiO2_ = (weight loss percentage)_total_ - (weight loss percentage)_APTMS_ - (weight loss percentage)_CNP_

Weight of APTMS in sample = (weight loss percentage APTMS*initial weight of (CNP+APTMS+FA) sample)/100

No. APTMS moles = (weight of APTMS/molecular weight of APTMS)

1 mole of APTMS forms 60.08 gm of SiO2

Weight of SiO2 in sample = No. APTMS moles*60.08

Weight of folic acid molecules = ([weight loss percentage folic acid + SiO2x initial weight]/100) – weight of SiO2

Total folic acid molecules in sample = (weight of folic acid molecules/molecular weight of folic acid)*N_A_

No. folic acid molecules per particles = (total folic acid molecules in sample)/(#CNP particles)
